# Design and rationale of the ATHENA study – A 12-month, multicentre, prospective study evaluating the outcomes of a de novo everolimus-based regimen in combination with reduced cyclosporine or tacrolimus versus a standard regimen in kidney transplant patients: study protocol for a randomised controlled trial

**DOI:** 10.1186/s13063-016-1220-9

**Published:** 2016-02-17

**Authors:** Claudia Sommerer, Barbara Suwelack, Duska Dragun, Peter Schenker, Ingeborg A. Hauser, Björn Nashan, Friedrich Thaiss

**Affiliations:** Nephrology Unit, University Hospital Heidelberg, Heidelberg, Germany; Department of Medicine D, Division of General Internal Medicine, Nephrology and Rheumatology, University Hospital of Münster, Münster, Germany; Department of Nephrology and Intensive Care Medicine, Charité Universtätsmedizin Berlin, Berlin, Germany; Department of General, Visceral and Transplant Surgery, University Hospital Knappschaftskrankenhaus Bochum, Ruhr-University Bochum, Bochum, Germany; Department of Nephrology, J.W. Goethe-University, Frankfurt, Germany; Department of Hepatobiliary Surgery and Transplantation, University Medical Center Hamburg-Eppendorf, Hamburg, Germany; III. Medical Clinic/Nephrology, Universitätsklinikum Hamburg-Eppendorf, Hamburg, Germany

**Keywords:** Everolimus, Renal function, Kidney transplantation

## Abstract

**Background:**

Immunosuppression with calcineurin inhibitors remains the mainstay of treatment after kidney transplantation; however, long-term use of these drugs may be associated with nephrotoxicity. In this regard, the current approach is to optimise available immunosuppressive regimens to reduce the calcineurin inhibitor dose while protecting renal function without affecting the efficacy. The ATHENA study is designed to evaluate renal function in two regimens: an everolimus and reduced calcineurin inhibitor-based regimen versus a standard treatment protocol with mycophenolic acid and tacrolimus in de novo kidney transplant recipients.

**Method/Design:**

ATHENA is a 12-month, multicentre, open-label, prospective, randomised, parallel-group study in de novo kidney transplant recipients (aged 18 years or older) receiving renal allografts from deceased or living donors. Eligible patients are randomised (1:1:1) prior to transplantation to one of the following three treatment arms: everolimus (starting dose 1.5 mg/day; C0 3–8 ng/mL) with cyclosporine *or* everolimus (starting dose 3 mg/day; C0 3–8 ng/mL) with tacrolimus *or* mycophenolic acid (enteric-coated mycophenolate sodium at 1.44 g/day or mycophenolate mofetil at 2 g/day) with tacrolimus; in combination with corticosteroids. All patients receive induction therapy with basiliximab. The primary objective is to demonstrate non-inferiority of renal function (eGFR by the Nankivell formula) in one of the everolimus arms compared with the standard group at month 12 post transplantation. The key secondary objective is to assess the incidence of treatment failure, defined as biopsy-proven acute rejection, graft loss, or death, among the treatment groups. Other objectives include assessment of the individual components of treatment failure, incidence and severity of viral infections, incidence and duration of delayed graft function, incidence of indication biopsies, slow graft function and wound healing complications, and overall safety and tolerability. Exploratory objectives include evaluation of left ventricular hypertrophy assessed by the left ventricular mass index, evolution of human leukocyte antigen and non-human leukocyte antigen antibodies, and a cytomegalovirus substudy.

**Discussion:**

As one of the largest European multicentre kidney transplant studies, ATHENA will determine whether a de novo everolimus-based regimen can preserve renal function versus the standard of care. This study further assesses a number of clinical issues which impact long-term outcomes post transplantation; hence, its results will have a major clinical impact.

**Trial registration:**

Clinicaltrials.gov: NCT01843348, date of registration – 18 April 2013; EUDRACT number: 2011-005238-21, date of registration – 20 March 2012

**Electronic supplementary material:**

The online version of this article (doi:10.1186/s13063-016-1220-9) contains supplementary material, which is available to authorized users.

## Background

The evolution of immunosuppressive regimens over the past few decades has led to considerable improvement in acute rejection rates and short-term graft survival [1]. Patient and graft survival at 1 year post transplantation now exceed 95 % in the case of living donation and 90 % after deceased donation. However, the long-term outcomes post kidney transplantation do not show a similar trend of improvement. At 10 years, graft survival remains about 50 % after deceased donation in the US and in Europe, with approximately 30 % of patients returning to dialysis and one of four patients dying with a functioning graft [1, 2]. The lack of improvement in long-term outcomes is further reflected by the fact that the number of re-transplants among adult kidney transplant patients has remained almost unchanged over the last decade [1].

Currently, calcineurin inhibitors (CNIs), cyclosporine, and tacrolimus are the cornerstone of immunosuppressive therapy post kidney transplantation [1]. However, their long-term use may be associated with non-reversible nephrotoxicity, morphologically characterised by striped fibrosis, progressive arteriolar hyalinosis, and ischemic glomerulosclerosis, which is a well-recognised cause of morbidity in transplant patients [3–5]. Chronic allograft injury alone accounts for two thirds of kidney graft failures [6]. Clinical data has shown that lowering the dose of CNI can improve renal function [5–7]. In this regard, the focus should be on optimising the currently available immunosuppressive regimens with the aim of preserving long-term renal function while maintaining the efficacy [8]. Several studies with a reduced-dose CNI and everolimus regimen have shown that it maintained efficacy and preserved renal function (Table 1) [9–33]. In the large randomised A2309 trial, pre-emptive everolimus therapy was associated with a greater than 60 % reduction in cyclosporine exposure while preserving renal function with comparable efficacy to mycophenolic acid and standard-exposure cyclosporine in de novo kidney transplant patients [16, 17]. In the ASSET study, an everolimus-facilitated tacrolimus minimisation strategy achieved good renal function with an acceptable safety profile without compromising efficacy [23]. Moreover, everolimus exerts other non-immunosuppressive properties, including potential cardioprotective, anti-malignancy, and antiviral effects [34–41]. These non-immunosuppressive benefits further suggest that everolimus-based regimens may be a preferred approach as cardiovascular disease, malignancy, and infections account for nearly four out of the five deaths occurring with functioning grafts [6].

**Table 1:** Everolimus in kidney transplantation

Study	Patients and treatment	Design	Key results
B156 Nashan et al. [9]	*N* = 111 de novo patients EVR (3 mg/day) + basiliximab + steroids with either full-dose CsA (C0 125–250 ng/mL) *or* reduced-dose CsA (C0 50–100 ng/mL)	3-year, phase II, open-label, multicentre, randomised, parallel-group study	• Efficacy failure was significantly lower in the reduced-dose CsA group vs. the full-dose CsA group at month 6 (3.4 % vs. 15.1 %; *p* = 0.046), month 12 (8.6 % vs. 28.3 %; *p* = 0.012), and month 36 (17.2 % vs. 35.8 %; *p* = 0.032) • Mean CrCL (mL/min) was higher in the reduced-dose CsA group vs. the full-dose CsA group at month 6 (59.7 vs. 51.1; *p* = 0.009), month 12 (60.9 vs. 53.5; *p* = 0.007), and month 36 (56.6 vs. 51.7; *p* = 0.436)
B201	*N* = 588 de novo patients	3-year, randomised, multicentre, parallel-group study; 1-year, double-blind, double-dummy and 2-year, open-label	• At months 12 and 36, efficacy failure rates were similar for all groups (*p* = NS) • At month 36, creatinine values were higher in the EVR groups, requiring a protocol amendment that recommended lower CsA exposure • Incidence of CMV infection was significantly lower at month 12 (*p* = 0.001) and month 36 (*p* = 0.0001) in the EVR groups vs. the MMF group
Vitko et al. [10] Vitko et al. [11]	EVR 1.5 mg/day or EVR 3 mg/day or MMF 2 g/day; all with standard CsA and steroids		
B251 Lorber et al. [12]	*N* = 583 de novo patients EVR 1.5 mg/day or EVR 3 mg/day or MMF 2 g/day; all with standard CsA and steroids	3-year, randomised, multicentre, parallel-group, study; 1-year, double-blind, double-dummy, and 2-year, open-label	• At months 12 and 36, primary efficacy failure rates were similar for all the arms (*p* = NS) • Incidence of antibody-treated acute rejection was significantly lower at month 12 (*p* = 0.01) and month 36 for the EVR 1.5 arm vs. the MMF arm (*p* = 0.014) • In a subgroup analysis, CsA dose reduction in the EVR arms resulted in improved renal function
A2306 Vitko et al. [13] Tedesco-Silva et al. [14]	*N* = 237 de novo patients EVR 1.5 mg/day or EVR 3 mg/day; both with low-dose CsA ± steroids	1-year, multicentre, randomised, open-label, parallel-group study	• Median serum creatinine levels were similar for both the EVR arms (month 6, 133 vs. 132 μmol/L; month 12, 131 vs. 130 μmol/L) • At month 6 and month 12, efficacy failure rates were similar for both arms (*p* = NS)
A2307 Vitko et al. [13] Tedesco-Silva et al. [14]	*N* = 256 de novo patients EVR 1.5 mg/day or EVR 3 mg/day; both with low-dose CsA + basiliximab induction ± steroids	1-year, multicentre, randomised, open-label, parallel-group study	• Median serum creatinine levels were similar for both the EVR arms (month 6, 130 μmol/L in both arms; month 12, 129 vs. 128 μmol/L) • At month 6 and month 12, efficacy failure rates were similar for both arms (*p* = NS)
US09 Chan et al. [15]	*N* = 92 de novo patients Low-dose tacrolimus vs. standard-dose tacrolimus; both with EVR 1.5 mg/day + steroids + basiliximab	6-month, prospective, multicentre, open-label, randomised, parallel-group, exploratory study	• No significant difference in mean serum creatinine between EVR with either low- or standard-dose tacrolimus treatment groups at 6 months (112 vs. 127 μmol/L; *p* = 0.114) • Mean eGFR rate was high and comparable between the EVR with low- or standard-dose tacrolimus groups (75.3 vs. 72.5 mL/min, *p* = 0.466) • BPAR and efficacy failure rates were low and comparable for both treatment arms
A2309 Tedesco-Silva et al. [16] Cibrik et al. [17]	*N* = 833 de novo patients EVR (1.5 mg/day, C0 3–8 ng/mL or 3 mg/day, C0 6–12 ng/mL) + reduced CsA vs. MPA + standard CsA	24-month, phase IIIb, multicentre, randomised, open-label, non-inferiority study	• At month 24, composite efficacy failure rates were 32.9 %, 26.9 %, and 27.4 % in the EVR 1.5 mg, EVR 3 mg, and MPA groups, respectively • Mean eGFR rate (MDRD; mL/min/1.73 m^2^) at month 24 was 52.2, 49.4, and 50.5 in the three arms, respectively
ZEUS (2418) Budde et al. [18] Budde et al. [19]	*N* = 300 de novo patients After initial immunosuppression with CsA + EC-MPS + steroids, patients at 4.5 months post transplant are randomised (1:1) to either continue the same regimen or switch to EVR (C0 6–10 ng/mL) + EC-MPS + steroids	12-month, phase IV, prospective, multicentre, open-label, randomised study with additional 48-month follow-up	• Adjusted mean cGFR was significantly higher at month 12 (+9.8 mL/min/1.73 m^2^; *p* < 0.0001) and at 5 years (+5.3 mL/min/1.73 m^2^; *p* <0.001) in the EVR group vs. the CsA group
CALLISTO (A2420) Albano et al. [20] Dantal et al. [21]	*N* = 139 de novo patients Immediate EVR treatment (day 1 post transplant; C0 3–8 ng/mL) vs. delayed EVR (week 5; C0 3–8 ng/mL). All patients also received low CsA, anti-IL-2 receptor induction therapy, and steroids	12-month, prospective, multicentre, open-label study	• Primary composite efficacy failure at month 3 occurred in 55.4 % patients in the immediate EVR group vs. 63.5 % in the delayed group (*p* = 0.387) while at month 12 the rates were 64.6 % and 66.2 %, respectively (*p* = 0.860) • At month 12, median eGFR values were 48 and 49 mL/min/1.73 m^2^ in the immediate EVR and delayed EVR groups, respectively • Incidence of DGF and wound healing complications were similar between the treatment groups
CERTES (A2419)/LATAM (A2423) Novoa et al. [22]	*N* = 119; A2419 de novo patients *N* = 51; A2423 de novo patients Initial treatment with EVR (C0 3–8 ng/mL) + CsA + basiliximab induction + steroids; randomisation (1:1) at 3 months to either continue the same regimen with CsA reduction (C2 300–500 ng/mL in A2419 and 350–450 ng/mL in A2423) or to start CsA elimination (by month 4 in A2419 and by month 6 in A2423) with EVR (C0 8–12 ng/mL)	12-month, multicentre, prospective, randomised, open-label study	• At month 12, eGFR rates were significantly higher in the CsA-elimination group vs. the CsA-minimisation group (68.3 vs. 63.6 mL/min/1.73 m^2^, *p* = 0.0289) • Post randomisation, the incidence of efficacy failure (BPAR, graft loss, death, loss to follow-up) at 12 months was comparable in the two groups: 18.9 % in the CNI-elimination group vs. 17.5 % in the CNI-minimisation group (*p* = NS)
ASSET (A2426) Langer et al. [23]	*N* = 228 de novo patients EVR (C0 3–8 mg/mL) with tacrolimus (C0 4–7 ng/mL) up to month 3; from month 4 either continue the same low-tacrolimus dose or start very low-tacrolimus dose (C0 1.5–3 ng/mL)	12-month, open-label, randomised study	• At month 12, mean eGFR was higher in the very low-tacrolimus group vs. the low-tacrolimus group (difference: 5.3 mL/min/1.73 m^2^; *p* = NS) • Incidence of BPAR from month 4 to month 12 was non-inferior (*p* = 0.0014) for the very low-tacrolimus group vs. the low-tacrolimus group (2.7 % vs. 1.1 %) • The incidence of NODM from month 4 to month 12 was numerically lower in the very low-tacrolimus group vs. the low-tacrolimus group (2.7 % vs. 8.6 %; *p* = 0.086).
APOLLO (DE02) Budde et al. [24] Budde et al. [25]	*N* = 93 maintenance patients (≥6 months post transplant) EVR (C0 6–10 ng/mL) + EC-MPS ± steroids vs. standard CNI (CsA C0 80–150 ng/mL or tacrolimus C0 5–10 ng/mL) + EC-MPS ± steroids	12-month, open-label, prospective, multicentre study with follow-up at month 60	• Mean time post transplant was 83.5 months with EVR vs. 70.1 months with CNI • Adjusted mean eGFR values (Nankivell, mL/min/1.73 m^2^) were numerically higher with EVR vs. CNI at month 12 (61.6 vs. 58.8; *p* = NS) and at month 60 (63.0 vs. 57.9; *p* = NS) • Using the MDRD formula, adjusted eGFR at month 12 was significantly higher (+4.9 mL/min/1.73 m^2^) with EVR vs. CNI (*p* = 0.030) • At month 60, for patients who remained on the study drug, mean eGFR was significantly higher with EVR vs. CNI (71.6 vs. 60.6; *p* = 0.005)
EVEREST (IT02) Salvadori et al. [26] Ponticelli et al. [27]	*N* = 285 de novo patients Standard EVR (C0 3–8 ng/mL) with low CsA (C2 350–500 ng/mL) vs. high EVR (C0 8–12 ng/mL) with very low CsA (C2 150–300 ng/mL)	6-month, multicentre, randomised, open-label, parallel-group study with follow-up at 12 months and an extension to 24 months	• Death-censored graft survival was significantly lower with standard EVR vs. the high EVR arm at month 6 (90.2 % vs. 97.9 %, *p* = 0.007) and at month 24 (87.4 % vs. 94.4 %, *p* = 0.048) • No significant difference between groups at months 6 and 24 for mean serum creatinine levels and incidence of BPAR
A1202 Takahashi et al. [28]	*N* = 122 de novo patients EVR (C0 3 to 8 ng/mL) + reduced-dose CsA vs. MMF (2 g/day) + standard-dose CsA. All patients receive basiliximab and steroids	12-month, phase III, multicentre, randomised, open-label, parallel-group, non-inferiority study	• 52 % reduction in CsA exposure was achieved in the EVR group at month 12 • At month 12, EVR with reduced CsA exposure was non-inferior to the MMF group for composite efficacy failure (11.5 % vs. 11.5 %) • Median eGFR at month 12 was comparable between the EVR arm vs. the MMF arm (58.00 vs. 55.25 mL/min/1.73 m^2^; *p* = 0.063)
ASCERTAIN (A2413) Holdaas et al. [29]	*N* = 398 maintenance patients Patients ≥6 months post transplant and receiving CNI ± MPA/azathioprine ± steroid randomised to either continue the same regimen (control arm) or switch to a CNI-elimination (EVR C0 8–12 ng/mL), or a CNI-minimisation by 70–90 % (EVR C0 3–8 ng/mL) regimen	24-month, phase IV, multicentre, prospective, randomised, open-label, parallel-group study	• At month 24, mean mGFR was comparable for all the three arms (*p* = NS) • Post-hoc analyses showed that patients with baseline CrCl >50 mL/min had a significantly greater increase in mGFR after CNI-elimination vs. the control arm (difference 11.4 mL/min/1.73 m^2^, *p* = 0.017) • Study drug discontinuation was significantly high in the CNI-elimination and CNI-minimisation arms vs. the control arm
SOCRATES (A2421) Chadban et al. [30]	*N* = 126 de novo patients Initial treatment with CsA + EC-MPS + steroids for the first 14 days post transplant then either continue the same regimen (control arm) or switch to + steroids + EC-MPS and CNI withdrawal, or EVR (C0 6–10 ng/mL) + CsA reduction + steroid and EC-MPS withdrawal	36-month, prospective, open-label, randomised controlled trial	• The steroid withdrawal arm was prematurely terminated due to the high rate of discontinuations • At month 12, EVR with CNI-withdrawal was non-inferior to the control arm for mean eGFR (65.1 vs. 67.1 mL/min/1.73 m^2^; *p* = 0.026) • Patients in the EVR with CNI-withdrawal group experienced a higher rate of BPAR vs. the control group (31 % vs. 13 %, *p* = 0.048)
MECANO (NL02) Bemelman et al. [31]	*N* = 113 maintenance patients Initial treatment with CsA + EC-MPS + steroids + basiliximab induction followed by randomisation at 6 months to start either CsA + MPA elimination, or MPA + CsA elimination, or EVR + CsA and MPA elimination; with steroids	24-month, prospective, open-label, randomised, multicentre study	• Post conversion, acute rejection rates were 3 % in the CsA group, 22 % in the MPA group, and 0 % in the EVR group (*p* <0.009) • Mean serum creatinine values were significantly lower at the latest follow-up (14 ± 5 months after transplantation) in the EVR arm vs. the CsA group
CENTRAL (ASE01) Mjörnstedt et al. [32]	*N* = 204 de novo patients Initial treatment with CsA + EC-MPS + steroids + basiliximab induction followed by randomisation at 7 weeks post transplant to either continue the same regimen, or convert to EVR (C0 6–10 ng/mL) + EC-MPS	36-month, open-label, parallel-group study	• From week 7 to month 12, change in mGFR was significantly greater with EVR vs. the CsA arm (4.9 vs. 0.0 mL/min; *p* = 0.012; ANCOVA). • No differences in graft or patient survival for both the groups • The 12-month incidence of BPAR was significantly high in the EVR arm vs. the CsA arm (27.5 % vs. 11.0 %; *p* = 0.004)

*ANCOVA* analysis of covariance, *BPAR* biopsy-proven acute rejection, *C0* trough levels, *C2* two hours post-dose, *cGFR* calculated glomerular filtration rate, *CMV* cytomegalovirus, *CNI* calcineurin inhibitors, *CrCl* creatinine clearance, *CsA* cyclosporine, *DGF* delayed graft function, *EC-MPS* enteric-coated mycophenolate sodium, *eGFR* glomerular filtration rate, *EVR* everolimus, *IL* interleukin, *MDRD* modification of diet in renal disease, *mGFR* measured glomerular filtration rate, *MMF* mycophenolate mofetil, *MPA* mycophenolic acid, *NODM* new-onset diabetes mellitus, *NS* not significant, *vs.* versus

The ATHENA trial is designed to further increase our knowledge and seek answers relating to the use of everolimus in CNI minimisation protocols in *de novo* kidney transplant patients. The ATHENA study assesses the change in renal function at 12 months post transplant as the primary objective. The design of the trial is described here.

## Methods/Design

### Study design

**Fig. 1:**
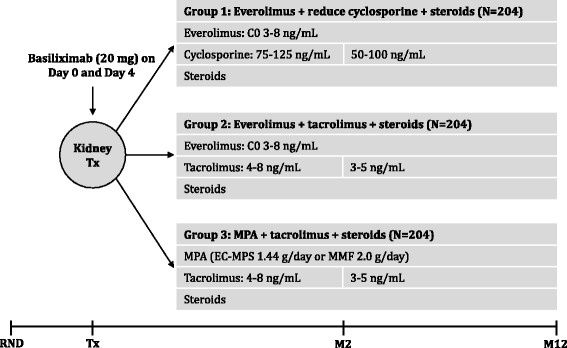
Study design. Steroid dose will be at least 5 mg prednisolone or equivalent, according to centre practice. *EC-MPS* enteric-coated mycophenolate sodium. *M* month, *MMF* mycophenolate mofetil, *MPA* mycophenolic acid, *RND* randomisation, *Tx* transplantation

ATHENA (Clinicaltrials.gov: NCT01843348; EUDRACT number: 2011-005238-21) is a 12-month, multicentre, randomised, international, prospective, controlled, open-label study with three parallel treatment groups in de novo kidney transplant recipients receiving renal allografts from deceased or living donors (protocol version 3, 29 July 2014). Eligible patients are randomised before transplantation using a validated system to ensure an unbiased treatment assignment in a 1:1:1 ratio to receive either everolimus with a reduced dose of cyclosporine, or everolimus with tacrolimus, or a standard regimen of mycophenolic acid with tacrolimus (Fig. 1). All patients receive induction therapy with basiliximab and maintenance steroids. At the time of randomisation, patients are stratified based on the donor type (living donor, deceased standard criteria donor, or deceased expanded criteria donor) and the participation of the recipient in the European Senior Program. The study protocol and the proposed informed consent form were reviewed and approved by the national institutional review boards or independent ethics committees at each centre and the federal institute for drugs and medical devices (Additional file 1). Written informed consent was obtained from all patients. The clinical study was designed and is conducted in accordance with the ethical principles laid down in the Declaration of Helsinki.

### Study population

The study population comprises de novo adult patients receiving a primary or secondary kidney transplant from a deceased or living donor. In the case of second kidney transplants, patients could be enrolled only if the first graft loss is due to non-immunological reasons. Patients are not eligible for the study if they are recipients of an ABO-incompatible transplants, have pre-existing donor-specific antibodies (DSA), or have an organ cold ischemia time longer than 30 h. Patients with pre-existing human leukocyte antigen (HLA)-antibodies not directed against the donor and less than 20 % panel reactivity at the time of transplant were included in the study. Detailed inclusion and exclusion criteria are shown in Table 2.

**Table 2:** Key inclusion and exclusion criteria

Key inclusion criteria	
•	Male or female renal allograft recipients aged18 years or older
•	Recipients of a primary or secondary kidney transplant from a deceased or living unrelated/related donor
•	Written informed consent to participate in the study
•	Cold ischemia time below 30 hours
•	Female patients who are menstruating and capable of conceiving must test negative for pregnancy before study enrolment and during the conduct of the study
Key exclusion criteria	
•	Multi-organ transplant recipients
•	Graft loss due to immunological reasons in the first year after transplantation (in case of secondary transplantation)
•	ABO-incompatible transplants
•	A current panel reactive antibody level of >20 % (within 4 months before enrolment) or positive Luminex test for any donor antigen
•	Existing antibodies against the HLA-type of the receiving transplant (known to the investigator at the time of transplantation)
•	History of malignancy during the last 5 years, except squamous or basal cell carcinoma of the skin, renal cell carcinoma ≤ T1N0M0, prostate adenocarcinoma ≤ T1N0M0, and adenocarcinoma of the thyroid
•	Thrombocytopenia or leukopenia, uncontrolled hypercholesterolemia, or hypertriglyceridemia
•	Pregnant or nursing (lactating) women Women of child-bearing age, unless they are using effective methods of contraception

*HLA* human leukocyte antigen

### Study objectives

The primary objective at month 12 post transplantation is to demonstrate non-inferiority in renal function assessed by the glomerular filtration rate (Nankivell formula) [41] in at least one of the everolimus treatment regimens compared with the standard treatment group receiving mycophenolic acid and tacrolimus. The key secondary objective at month 12 is to evaluate the incidence of treatment failure defined as biopsy-proven acute rejection (BPAR), graft loss, or death among the treatment groups. Other objectives include assessment of individual components of treatment failure, incidence and severity of viral infections (cytomegalovirus (CMV), BK-virus (BKV)), incidence and duration of delayed graft function (DGF), incidence of indication biopsies, incidence of slow graft function, incidence of wound healing complications, and duration of healing. Incidence of viral infections (CMV and BKV) and changes to the viral load are closely monitored throughout the study. In addition, a patient subgroup analysis of CMV-specific T-cells and NK-cells is conducted in a central laboratory. Overall, the safety objectives include assessment of adverse events (AEs) and serious AEs (SAEs), infections and discontinuations due to AEs, and laboratory abnormalities. Exploratory objectives include evaluation of the incidence of HLA-antibodies and among those DSA and non-HLA antibodies (AT_1_R, ET_A_R) by treatment group and its association with acute rejection. HLA-antibodies including DSA were analysed using single antigen Luminex technology at baseline and month 12. In addition, left ventricular (LV) changes are measured by LV hypertrophy (LVH) assessment by echocardiography measurements. Detailed objectives of the study are outlined in Table 3.

**Table 3:** Objectives of the ATHENA study

Primary objective	
•	To demonstrate non-inferiority in renal function (estimated GFR by the Nankivell formula) in at least one of the everolimus arms compared with the standard regimen at month 12 post transplantation
Key secondary objectives	
•	To assess the incidence of treatment failure (composite of biopsy-proven acute rejection, graft loss, or death) at month 12 post transplantation
Other secondary objectives	
To evaluate the following:	
•	GFR by different formulae (CKD-EPI, Cockcroft-Gault and MDRD)
•	Incidence of individual efficacy endpoints: biopsy-proven acute rejection, graft loss, and death
•	Incidence and severity of viral infections (CMV, BKV)
•	Incidence and duration of delayed graft function
•	Incidence of slow graft function defined as serum creatinine >3.0 mg/dL at day 5
•	Incidence of wound healing complications related to the surgery and the duration of healing
•	Overall safety and tolerability (incidence of AEs and serious AEs, infections, discontinuation due to AEs, and laboratory abnormalities) at month 12 post transplantation
Exploratory objectives	
•	To compare HLA- and non-HLA antibody evolution at baseline and month 12 post transplantation
•	To evaluate left ventricular hypertrophy (assessed by LV mass index) and diastolic dysfunction
•	The incidence of donor-specific antibodies by treatment group, and its association with acute rejection
•	Analysis of general immunomodulatory effects on lymphocyte subpopulations and on the incidence and antigen-specific immune control of CMV infections

*AEs* adverse events, *BKV* BK-virus, *CKD-EPI* Chronic Kidney Disease Epidemiology Collaboration, *CMV* cytomegalovirus, *GFR* glomerular filtration rate, *HLA* human leukocyte antigen, *LV* left ventricular, *MDRD* modification of diet in renal disease

### Immunosuppression

All patients receive induction therapy with basiliximab dosed at 20 mg intravenously on the day of transplantation and on day 4 post transplantation, as per label recommendations. Patients are randomised to receive everolimus at an initial dose of 1.5 mg/day with a reduced dose of cyclosporine and 3 mg/day with tacrolimus within the first 24 h post transplantation. Thereafter, the dose of everolimus is adjusted to the target trough concentration of 3–8 ng/mL throughout the study period. Treatment with CNI, tacrolimus, or cyclosporine, is initiated within the first 24 h post transplantation, and the dose of CNI is adjusted to maintain the target trough levels, as shown in Fig. 1. In the control group, patients receive mycophenolic acid at a dose of 1.44 g/day enteric-coated mycophenolate sodium or at a dose of 2 g/day mycophenolate mofetil with a standard dose of tacrolimus. Dose adjustments and interruptions are allowed for tolerability reasons as defined in the protocol and are recorded. All patients receive corticosteroids at a minimum dose of 5 mg/day prednisolone or equivalent until month 12. Acute rejections are treated according to local practice and physicians’ discretion. All patients who prematurely withdraw from the study are provided with follow-up medical care/referred for appropriate ongoing care, as per the local practice.

### Concomitant medication

Mandatory CMV prophylactic therapy with valganciclovir is recommended for at least 3 months in the case of high- to moderate-risk patients (CMV-positive donor/CMV-negative recipients or CMV-positive donor/CMV-positive recipients). All patients receive prophylactic treatment for *Pneumocystis jirovecii* pneumonia with trimethoprim/sulfamethoxazole for a period of 6 months. All medications and significant non-drug therapies administered after the initiation of the study drug are recorded.

### Data collection

Patient visits are scheduled at baseline, and at months 1, 3, 6, 9, and 12 post transplantation. A detailed list of all study assessments and visits is shown in Table 4. Patients who discontinue the study drug and those who prematurely withdraw from the study are scheduled for a visit and all the assessments listed for visit 6 are performed.

**Table 4:** Assessment schedule

	12-month study period						
Month	Baseline	1	3	6	9	Premature end of treatment/withdrawal	12
Visit	1	2	3	4	5	6	7
Enrolment							
Informed consent	X						
Inclusion/Exclusion	X						
Randomisation	X						
Demography	X						
General medical history	X						
Transplantation information	X						
Viral serology	X						
Pregnancy test (β-HCG)	X						
Interventions							
Trough levels (everolimus, cyclosporine, tacrolimus)		X	X	X	X	X	X
Assessments							
Physical examination	X					X	X
Vital signs	X	X	X	X	X	X	X
Study medication check		X	X	X	X	X	X
Haematology/Biochemistry	X	X	X	X	X	X	X
Urinalysis		X	X	X	X	X	X
Viral assessments		X	X	X	X	X	X
Serum for non-HLA antibodies and DSA	X			X		X	X
Echocardiography (LVH)	X					X	X
Protocol renal allograft biopsy^a^	X			X			X
Biomarker assessments^b^	X	X				X	X
CMV substudy^b^	X					X	X
Wound healing complications	As necessary						
Rejection episodes							
Indicated renal allograft biopsy							
Dialysis							
AEs/SAEs/Infections/Comments							
Concomitant therapy							
Immunosuppressive therapy							
End of treatment						X	X
End of study						X	X

*β-HCG* human chorionic gonadotropin, *AE* adverse events, *DSA* donor-specific antibodies, *HLA* human leukocyte antigen, *LVH* left ventricular hypertrophy, *SAE* severe adverse events

^a^Not mandatory. Can be performed according to centre practice

^b^Only in selected centres and patients

#### Renal function

Renal function is assessed by determining the glomerular filtrate rate using serum creatinine values according to the Nankivell formula [42] and used as the primary outcome measure in the study. In addition, as a secondary efficacy variable, the glomerular filtration rate is calculated using the Cockcroft-Gault method [43], the modification of diet in renal disease (MDRD) method [44–46], and the Chronic Kidney Disease Epidemiology Collaboration (CKD-EPI) method [47]. Serum creatinine levels are analysed using venous blood drawn and analysed in the local laboratory.

#### Outcome measures

BPAR is defined as rejections that are acute and proven by biopsy. The time to BPAR is the time from randomisation to the date of first documented BPAR. Graft loss is defined as a failure to discontinue dialysis or if the patient undergoes graft nephrectomy. Overall survival is defined as time from date of randomisation to death due to any cause. Delayed graft function is defined as the need for dialysis within the first 7 days post transplantation excluding the first day, and the duration is defined from the first dialysis day up to the last. Slow graft function is defined as serum creatinine >3.0 mg/dL at day 5.

#### Kidney allograft biopsy

Optional allograft biopsies are performed intra-operatively at the time of transplantation and at month 12. A control biopsy at month 6 may be performed according to centre practice. In all cases of suspected acute rejection, a graft biopsy is performed prior to, or within 24 h of initiation of anti-rejection therapy. All biopsies are read by the local pathologist according to the updated Banff 2009 criteria. Optional biopsies are assessed for the presence of interstitial fibrosis and tubular atrophy using the Banff 2007 criteria.

#### Non-HLA and HLA antibodies

The presence and evolution of non-HLA and HLA antibodies, and among those especially DSA antibodies in the serum, is evaluated at a central laboratory. Blood samples (5 mL) are collected for all patients at baseline, month 6, and month 12.

#### LVH and diastolic dysfunction

The echocardiographic analysis included assessments of the end-diastolic interventricular septum, LV end-diastolic posterior wall thickness, LV end-diastolic diameter, LV end-systolic diameter, LV end-diastolic volume, LV end-systolic volume, LV ejection fraction, relative wall thickness, and LV mass also expressed as LV mass index [48]. LVH was defined as an LV mass index exceeding 110 g/m^2^ in women and 125 g/m^2^ in men, and a value of 0.44 was taken as cut-off point for abnormal relative wall thickness [49]. Diastolic dysfunction was assessed and graded according to the guidelines of the American Society of Echocardiography [50].

#### CMV substudy

In this optional substudy, 4.7 mL of whole blood is drawn into a lithium heparin-containing tube at baseline and at month 12 or at the end of study/treatment, and shipped on the same day to a central laboratory at 4 °C. This substudy prospectively monitors the incidence of viraemia by analysis of viral load, CMV-specific T-cell frequency, phenotype and functionality, and regulatory T-cells, and T- and NK-cell subsets.

#### Data Monitoring Board

An external and independent Data Safety Monitoring Board was instituted before the start of the study. The board reviews safety-related issues on an ongoing basis and is entitled to make recommendations for changes in study conduct.

### Statistical analysis

The primary efficacy variable, i.e., renal function at month 12 after randomisation between the treatment groups will be compared with the analysis of covariance (ANCOVA) model, using the treatment and centre as factors, and the estimated glomerular filtration rate at baseline as a covariate. Missing estimated glomerular filtration values will be handled within the ANCOVA analysis by multiple imputations by the last available post-baseline observation carried forward (LOCF) approach. Assuming a common dropout rate of 20 %, a sample size of 612 patients (204 patients in each treatment arm) is required in the study, so as to have at least 80 % power to demonstrate non-inferiority (2.5 % margin, one-sided *t* test) for the primary endpoint.

The primary analysis is based on the full analysis set that consists of all patients who receive at least one dose of the study drug. The per-protocol set includes all patients in the full analysis set who do not have any major deviations from the protocol procedures that may impact the study outcomes. The safety set consists of all patients who receive at least one dose of the study drug and had at least one post-baseline safety assessment.

## Discussion

ATHENA is one of the largest European multicentre kidney transplant studies. It is the first study evaluating the non-inferiority of renal function as a primary objective in a de novo everolimus-based immunosuppressive protocol, and will determine whether an everolimus-based regimen can preserve renal function versus the current standard of care. The study will also provide insights into the evolution of HLA and non-HLA antibodies, occurrence of viral infections post transplantation, surveillance of cardiovascular comorbidities, and the incidence of wound healing complications [51]. In addition, the trial explores a regimen of everolimus with reduced-dose cyclosporine while the dose of tacrolimus in the everolimus arm and the standard arm is the same. These unique features will further enable the study to provide a direct comparison between the two arms, which will in turn help in optimising the immunosuppressive protocols. As the study addresses a wide range of issues that clinicians face today, its results are awaited with interest.

### Trial status

The study is currently ongoing and actively recruiting patients across 27 sites, 15 centres in Germany and 12 centres in France. The study is expected to be complete by March 2016.

## Additional file

Additional file 1:
**List of ethics committees.** (PDF 184 kb)

## Abbreviations

AEs: adverse events
ANCOVA: analysis of covariance
BPAR: biopsy-proven acute rejection
C0: trough levels
cGFR: calculated glomerular filtration rate
CKD-EPI: Chronic Kidney Disease Epidemiology Collaboration
CMV: cytomegalovirus
CNI: calcineurin inhibitors
CrCl: creatinine clearance
CsA: cyclosporine
DGF: delayed graft function
DSA: donor-specific antibody
EC-MPS: enteric-coated mycophenolate sodium
eGFR: estimated glomerular filtration rate
EVR: everolimus
HLA: human leukocyte antigen
LV: left ventricular
LVH: left ventricular hypertrophy
MDRD: modification of diet in renal disease
mGFR: measured glomerular filtration rate
MMF: mycophenolate mofetil
MPA: mycophenolic acid
NODM: new-onset diabetes mellitus
NS: not significant
SAE: serious adverse events
