# Supplementary material for: Design and rationale of the ATHENA study – A 12-month, multicentre, prospective study evaluating the outcomes of a de novo everolimus-based regimen in combination with reduced cyclosporine or tacrolimus versus a standard regimen in kidney transplant patients: study protocol for a randomised controlled trial
Source: Trials. 2016 Feb 17;17:92. doi: 10.1186/s13063-016-1220-9 (PMC4756406; doi:10.1186/s13063-016-1220-9)
Supplement: Additional file 1: — List of ethics committees. (PDF 184 kb) [file 13063_2016_1220_MOESM1_ESM.pdf]

List of Ethics Committees

Lead Ethics Commission Germany and France

Hamburg Medical Association
Ethics Committee
Humboldtstr. 67a
22083 Hamburg
Tel: 040-202299240
Fax: 040-202299410

Committee to Protect People
Southwest and Overseas I and II
ARS Midi-Pyrénées
10 chemin du raisin
31050 TOULOUSE Cedex 9
Tel: 05.34.30.27.55
Fax: 05.34.30.27.38

Participating Ethics Committees

Ethics Committee at the Medical Faculty of the RWTH Aachen
Pauwelsstr. 30
52074 Aachen
Tel: 0241-8089963
Fax: 0241-8082012

State Office for Health and Social Affairs
Ethics Committee of Berlin
Fehrbelliner Platz 1
10707 Berlin
Tel: 030-90229 1226
Fax: 030-90283383

Ruhr-University Bochum
Medical school
Ethics Committee
Bürkle-de-la-Camp-Platz 1
44789 Bochum
Tel: 0234-3026421
Fax: 0234-3026426

Technical University Dresden
Medical Faculty Carl Gustav Carus
Ethics Committee
Fetscherstr. 74
01307 Dresden
Tel: 0351-4582992
Fax: 0351-4584369

University of Erlangen-Nuremberg
Medical School
Ethics Committee
Krankenhausstr. 12
91054 Erlangen
Tel: 09131-85 22210
Fax: 09131-85 26021

University Hospital Essen
Medical Faculty of the University of Duisburg-Essen
Ethics Committee
Robert-Koch-Straße 9-11
45147 Essen
Tel: 0201-723 3637
Fax: 0201-723 5837

Ethics Committee of the Faculty of Medicine of the Johann Wolfgang Goethe University
University Hospital
Theodor-Stern-Kai 7
60590 Frankfurt am Main
Tel: 069-63014597
Fax: 069-630183434

University of Freiburg
Ethics Committee
Engelberger Str. 21
79106 Freiburg
Tel: 0761-27072600
Fax: 0761-27072510

Ethics Committee of the Hannover Medical School
Carl-Neuberg-Str. 1
30625 Hannover
Tel: 0511-5329229
Fax: 0511-5325423

Ethics Committee of the Medical Faculty of Heidelberg
Alte Glockengießerei 11/1
69115 Heidelberg
Tel: 06221-338220
Fax: 06221-3382222

Ethics Committee of the Medical Faculty of the University of Kiel
Schwanenweg 20
24105 Kiel
Tel: 0431-5971809
Fax: 0431-5975333

Ethics Committee of the medical association of Rheinland Pfalz
Deutschhausplatz 3
55116 Mainz
Tel: 06131-2882263
Fax: 06131-2882266

University Hospital Tübingen
Ethics Committee at the Faculty of Medicine Tübingen
Gartenstraße 47
72074 Tübingen
Tel: 07071-2977661
Fax: 07071-295965

University Hospital Münster
Ethics Committee of the Medical Association of Westfalen-Lippe
Gartenstr. 210-214
48147 Münster
Tel: 0251-9292460
Fax: 0251-9292478
